# Systematic Review on Chinese Herbal Medicine Induced Liver Injury

**DOI:** 10.1155/2016/3560812

**Published:** 2016-08-29

**Authors:** Peng Zhang, Yongan Ye, Xianzhao Yang, Yuntao Jiao

**Affiliations:** ^1^The First Clinical Medical School, Beijing University of Chinese Medicine, Beijing, China; ^2^Department of Gastroenterology and Hepatology, Beijing University of Chinese Medicine Affiliated Dongzhimen Hospital, Beijing, China; ^3^Department of Infectious Disease, Beijing University of Chinese Medicine Affiliated Dongzhimen Hospital, Beijing, China

## Abstract

*Background*. In recent years, with the popularity of CHM, its hepatotoxicity has also been increasingly noticed. However, there are still veils on causative herbs and clinical characteristics.* Aim*. To systematically review data on CHM induced liver injury with particular focus on causative herbs and clinical characteristics.* Methods*. Using terms related to CHM and liver injury, PubMed and three Chinese electronic databases were searched, which was limited to the past 5 years. Publications meeting our eligibility criteria were included and further analyzed.* Results*. In total, 4 single herbs, 21 patent drugs, and 4 decoctions were reported to be of hepatotoxicity, with He-Shou-Wu being the most common one (65/114). Dang-Gui and other 5 herbs were the most common ingredients of patent drugs and decoctions. All patients were assessed using the RUCAM scale, with 26 being highly probable and 28 being probable. For these 54 cases, the latent period was 30 (47) days, and 81.48% were labeled as hepatocellular injuries. Most patients (96.3%) recovered, apart from the fact that one died and one is receiving liver transplantation.* Conclusions*. CHM should be used carefully for hepatotoxicity. Liver injury from CHM is similar to that from conventional medicines in clinical characteristics. Details about causative herbs should be illustrated, and more RUCAM should be used in future.

## 1. Introduction

Traditional Chinese medicine (TCM), originated in ancient China, has been widely used to treat diseases for thousands of years, using Chinese herbal medicine (CHM), acupuncture, moxibustion, and other body practices. In recent decades, TCM has been increasingly popular around the world [1–4]. As the main part of TCM, CHM is usually combined in formulas and taken orally as decoction, powders, and other forms, following TCM theories. Although CHM plays an important role in health care, more and more liver injury cases from CHM are reported. While the exact number is unavailable, nearly 20% of drug-induced liver injuries (DILI) were due to CHM in China [5, 6]. Therefore, it is of great importance to study CHM induced liver injury.

To learn CHM induced liver injury, some questions are inevitable, namely, which herbs can lead to liver injury specifically, when it will cause liver injury, and what the clinical characteristics are. Though some reviews [7–10] have been published to give detailed information, they did not take Chinese electronic database into account, in which large amounts of data about use and adverse events of CHM are found. While these reviews did put an emphasis on causality assessment by the Roussel Uclaf Causality Assessment Method (RUCAM) scale [11] and positive reexposure tests, they paid little attention to strict definition of hepatotoxicity. A low threshold of liver enzyme values may allow cases with nonspecific increases in.

To help clinicians and TCM practitioners know more about and avoid CHM induced liver injury, we systematically reviewed publications, giving a list of Chinese herbal medicines with possible hepatotoxicity and summarizing associated clinical characteristics. Since more attention has been paid to DILI in recent years and the diagnosis has also developed both in China and the world, our review focused on literature of the past 5 years.

## 2. Methods

### 2.1. Literature Search

Our review was planned and performed in conformity with* Cochrane Handbook for Systematic Reviews of Interventions* [12] and Preferred Reporting Items for Systematic Review and Meta-Analysis (PRISMA) statement [13], which was also published on the PROSPERO register, with a registration number being CRD42016036053. A literature search in PubMed database and three Chinese electronic databases, including China National Knowledge Infrastructure (CNKI), Wan Fang database, and VIP database, was independently carried out by two investigators, using the terms “herb^*∗*^,” “Chinese medicine,” “traditional medicine,” and “complementary and alternative medicine” and “liver injury,” “hepatotoxicity,” “liver disease,” and “hepatitis.” The maximal number of articles was obtained using terms in all possible combinations. The search was limited to English and Chinese language articles and restricted between 2011 and March 1, 2016.

### 2.2. Eligibility Criteria

Articles included have to meet the following criteria. (1) Studies on human subjects are included. (2) Liver injury is specifically induced by Chinese herbal medicines, which include single herbs, patent drugs, and decoctions made up of herbal ingredients. Herbs included should be usually used by TCM practitioners or officially listed in the Chinese Pharmacopoeia [14]. (3) Liver injury is defined as elevations of ALT above 5 times the upper limit of normal (ULN) and/or ALP above 2 times ULN. If ALT > 5ULN and ALP ≤ ULN or if both ALT and ALP are elevated, *R* ≥ 5, the liver injury is hepatocellular. If ALP > 2ULN and ALT ≤ ULN or if both ALT and ALP are elevated, *R* ≤ 2, the liver injury is cholestatic. If ALT > 5*N* and ALP >* N* and 2 < *R* < 5, the liver injury is mixed. (4) Causality assessment is done using the RUCAM scale [11], with a score no less than 3 points. If the pattern of liver injury is hepatocellular, a subtype of RUCAM for hepatocellular injury is used, and if it is cholestatic or mixed, a subtype for the cholestatic or mixed injury is performed.

### 2.3. Study Selection and Data Extraction

Included articles were independently reviewed by two authors, based on title/abstract firstly and full-text secondly. During the process of full-text selection, disagreements were resolved by discussion, and if an agreement could not be reached, a third author would make a decision. The following data were recorded: causative herbs, demographic information, regional distribution, primary diseases, usage and dosage, latent period, laboratory results, pattern of liver injury, causality assessment, reexposure results, and clinical outcomes.

### 2.4. Statistical Analysis

A descriptive analysis was used. Enumeration data was described with frequency distribution, while measurement data was described with centralized tendency. Normally distributed data was described as Mean ± Standard Deviation, while data obeying abnormal distribution was presented as Median (Interquartile Range). All statistical analyses were performed using SPSS software (version 20.0). Cases with incomplete clinical information were also included in this review, but only those with well-defined values for each parameter were included in the statistical analysis.

## 3. Results

### 3.1. Literature Selection and Characteristics

**Figure 1:**
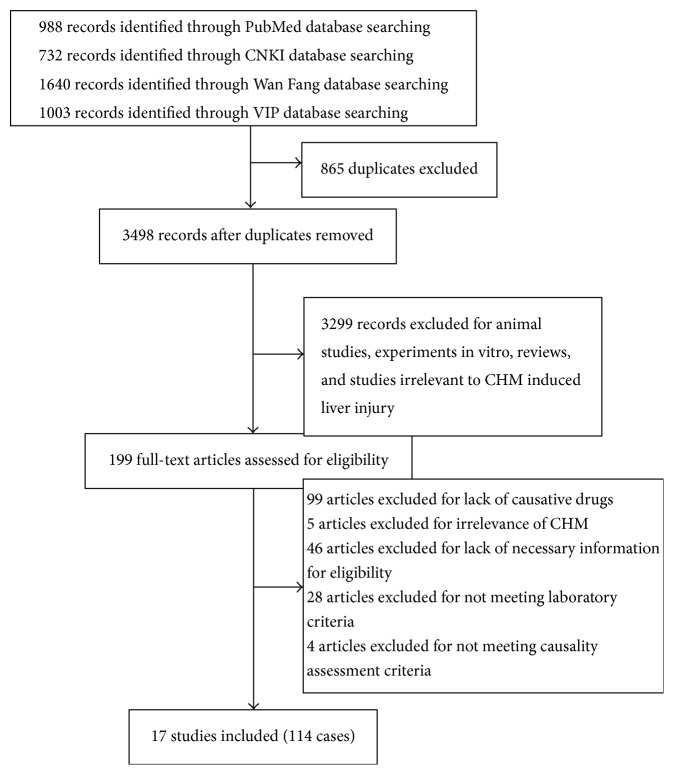
Flow chart of literature selection.

The initial search produced 4363 articles (Figure 1), of which 865 were excluded for duplicates and 3299 were eliminated as animal studies, experiments in vitro, reviews, and studies irrelevant to CHM. After further evaluation, 17 articles [15–31] fulfilled the eligibility criteria, of which 14 were case reports, 2 were case series, and 1 was cross-sectional study. 114 cases were included in total, of which 83 cases were from China, 26 were from Korea, 4 were from the United States, and 1 was from Japan. Detailed information of CHM, like locality and specific taxa, was not mentioned in all 17 articles, and only 4 of 17 described correct scientific names of herbs [18, 19, 21, 22]. Among six articles reporting liver injury attributed to Chinese patent drugs [15–17, 21, 23, 29], there was one [29] without detailed herbal ingredients, six without herbal contents, three [15, 23, 29] without recommended dosage and usage, and three [16, 23, 29] without brand names or manufacturers. Only 1 of 4 articles reporting decoctions mentioned detailed herbal dosage [30].

### 3.2. Identification of CHM with Reported Hepatotoxicity

**Figure 2:**
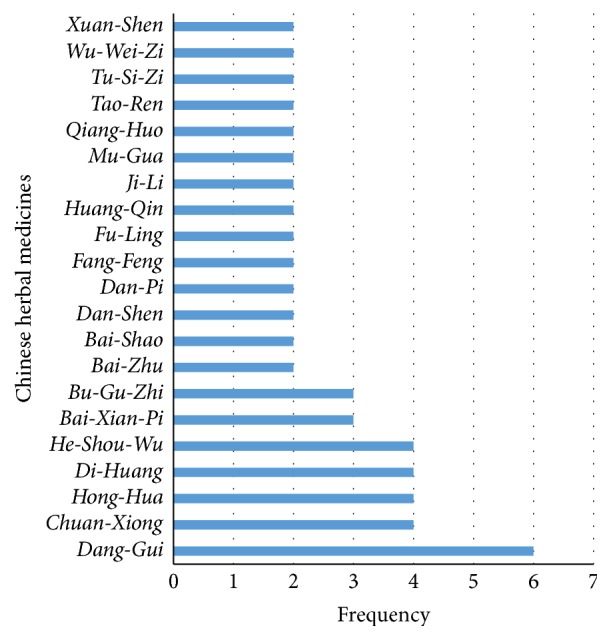
Frequency distribution of herbal ingredients of patent drugs and decoctions was performed, and herbs used more than once were given in the figure.

In total, 4 kinds of single herbs, 21 patent drugs, and 4 decoctions made up of multiple herbs were reported to have caused liver injury, including He-Shou-Wu [*Reynoutria multiflora* (Thunb.) Moldenke], Cang-Er-Zi [*Xanthium strumarium* subsp.* sibiricum* (Patrin ex Widder) Greuter], Huang-Yao-Zi (*Dioscorea bulbifera* L.), Lei-Gong-Teng (*Tripterygium wilfordii* Hook. f.), Yang Xue Sheng Fa Jiao Nang, Bai Dian Feng Jiao Nang, Xiao Yin Pian, Qu Bai Ba Bu Pian, Bu Shen Sheng Fa Tang, Ze Qi Chong Ji, Xian Ling Gu Bao Jiao Nang, Gu Kang Jiao Nang, Zhuang Gu Jiao Nang, Ling Zhi Yi Shou Jiao Nang, Ling Zhi Jiao Nang, Hui Chun Ru Yi Jiao Nang, Ru Bi San, ShuXiong Jiao Nang, Zeng Sheng Ping, Long Bi Shu, Zhi Xue Jiao Nang, Move Free, Ban Tu Wan, Kamishoyosan, Qi Bao Mei Ran Wan, herbal extracts containing Hu-Ji-Sheng [*Viscum coloratum* (Kom.) Nakai] and Ye-Ge (Pueraria montana var. lobata (Willd.) Sanjappa & Pradeep), herbal tea containing Kelp, and two decoctions consisting of CHM. Detailed information is listed in Table 1. Of the total 114 cases, liver injury caused by He-Shou-Wu accounted for 65, which was the most common one in our review. With regard to primary diseases of included cases, dermatosis, grey hair, and alopecia took up 11/30, which was the largest proportion. Other applications involved were osteoarthrosis (5/30), health promotion (3/30), diabetes mellitus (2/30), and mammary gland disorders (2/30). In addition, a further analysis about ingredients of patent drugs and decoctions was performed. Totally, 72 kinds of herbs were involved in 4 decoctions and 8 patent drugs with detailed ingredients, reported in articles or the Chinese Pharmacopoeia, of which Dang-Gui [*Angelica sinensis* (Oliv.) Diels], He-Shou-Wu, Di-Huang [*Rehmannia glutinosa* (Gaertn.) DC.], Chuan-Xiong (*Ligusticum striatum* DC.), Hong-Hua (*Chelonopsis pseudobracteata* var.* rubra* C. Y. Wu & H. W. Li), Bai-Xian-Pi (*Dictamnus albus* L.), and Bu-Gu-Zhi [*Psoralea cordata* (Thunb.) Salter] were the most common ones. Details are shown in Figure 2.

### 3.3. Causality Assessment

Causality assessment is necessary for the diagnosis of CHM induced liver injury. While expert consensus opinion and the RUCAM scale are considered as preferred algorithms to establish causality in suspected herb-induced liver injury (HILI), the former one is not widely available since it is cumbersome, costly, and time consuming. RUCAM scale is much more widely used by clinicians and researchers, which is structured, quantitative, and validated for liver injury. Of the total 17 articles included, 7 directly used the RUCAM scale to assess causality, while the other 10 articles provided associated information, based on which RUCAM can be performed. Four articles [9, 25, 28, 31] explicitly excluded HEV by serology tests, and four [20, 22, 26, 30] demonstrated exclusion of viral hepatitis without specific mention of HEV. Fifty-six cases of one article [29], scored no less than 3 points, were not supplied with detailed information and cannot be graded by RUCAM. Other cases were grouped into different likelihood levels, with 26 being highly probable (score > 8), 28 being probable (6–8), and 4 being possible (3–5). In a further analysis, 54 cases with more than 6 points of RUCAM were identified, in which He-Shou-Wu, Huang Yao Zi, Move Free, Kamishoyosan, Qi Bao Mei Ran Wan, herbal extracts containing Hu-Ji-Sheng and Ye-Ge, herbal tea containing Kelp, and a decoction consisting of CHM were causative. Since these herbs were more likely to cause liver injury, more attention should be paid. Furthermore, one case attributed to He-Shou-Wu was reported with a positive reexposure result, which accorded with the criteria of reexposure [11], while another case caused by kamishoyosan did not supply detailed ALT level of the first time, which, consequently, could not be diagnosed as reexposure.

### 3.4. Clinical Characteristics

Patients' features and clinical characteristics of liver injury from CHM were summarized. Of the 58 cases with detailed information, all 54 with probable or highly probable causality grading were included. Thus, we focused on these 54 cases and showed their clinical characteristics. While 35 were male and 19 were female, there was an average age of 47.13 ± 12.24 years, ranging from 17 to 78. The latent period was 30 (47) days, which is consistent with DILI [38]. Nine of eleven cases with well-defined information of actual and recommended dosage were involved in excessive intake of CHM, indicating that excessive use may be related to the incidence of liver injury. Concerning laboratory parameters, the average serum levels of ALT and AST were 1246 (824.25) IU/L and 931.52 ± 598.36 IU/L, respectively, and the average serum level of ALP was 225.08 ± 170.66 IU/L. The high laboratory values may be due to our restriction criteria for hepatotoxicity, which helps eliminate unspecific liver enzymes' increases and substantiate causality at higher probability. Cases were further identified by the pattern of liver injury, consisting of forty-four (81.48%) of hepatocellular injuries, eight (14.81%) of mixed injuries, and two (3.7%) of cholestatic injuries. Most of patients recovered from CHM induced liver injury, with a percentage of 96.3%, apart from the fact that one died and one is receiving liver transplantation. Additional details are available in Table 2.

## 4. Discussion

DILI is one of the most common causes of hepatitis in the world. According to some studies, DILI accounted for about 11% of acute liver failure cases [39, 40]. As a result of popularity, liver injury induced by CHM is on the increase around the world. To help TCM practitioners avoid CHM induced liver injury and supply clinicians with more associated data, we systematically reviewed publications, focusing on developments in the recent 5 years.

Characteristics of included cases were summarized, while problems in the original papers were brought up. Detailed information about CHM, like locality, botanical classification, brand names, detailed contents, and usage and dosage, was unavailable in most publications, which is of great importance to improve the reliability of studies. CHM with reported hepatotoxicity were identified, of which He-Shou-Wu attracted our attention. He-Shou-Wu is one of the most popular CHM, officially listed in the Chinese Pharmacopoeia. According to Ben Cao Gang Mu (*Compendium of Materia Medica*), an ancient book recording therapeutic effects of CHM, He-Shou-Wu is usually used to treat alopecia and white hair by nourishing the liver and kidneys. In recent years, with the increasing use of it, numbers of hepatotoxicity cases have been reported [18, 19, 41, 42]. Although efforts have been made, the toxicity mechanism is still not fully elucidated. Probably, the hepatotoxicity is related to some bioactive compounds, anthraquinone derivatives [32, 43]. Based on TCM theory, processing is believed to be able to reduce the toxicity of herbs, including He-Shou-Wu, but both processed and unprocessed He-Shou-Wu were reported to cause liver injury in publications. The phenomenon does not necessarily mean that preparation is of no use to hepatotoxicity. Since the use of the processed one is much more common than the unprocessed one and the hepatotoxicity can be affected by many other factors, the relationship between processing and hepatotoxicity cannot be concluded for now. Our review also indicated that hepatotoxicity was much more common with dermatosis and osteoarthrosis, which reminds dermatologists or orthopedists of avoiding those recorded herbs. Since people increasingly focus on health care, CHM used for health promotion also should be taken carefully.

It is worth noting that formulae consisting of multiple herbs, including patent drugs and decoctions, prevail in CHM with potential hepatotoxicity. In the view of TCM, liver injury from these drugs and decoctions is often related to some ingredients with hepatotoxicity. After a review of related literature, in our results, He-Shou-Wu, Bai-Xian-Pi, and Bu-Gu-Zhi have been reported to be of hepatotoxicity [10, 44], while Dang-Gui, Di-Huang, Chuan-Xiong, and Hong-Hua have not been, among which Dang-Gui, Di-Huang, and Hong-Hua have been reported to be with hepatoprotective effect [45–47]. These three herbs are often used in TCM prescriptions, especially used for skin and gynecological diseases, which may account for their high frequency. TCM practitioners always use a prescription to treat diseases, which blends together a number of herbs with specific functions. Sometimes, prescription compatibility may also play an important role in attenuating adverse events of CHM [48]. It is definitely necessary for clinicians to supply detailed ingredients and contents in articles as far as possible. However, during literature selection, lots of articles were excluded for lack of detailed information of multiple ingredients, which is the same as situation on the online websites, LiverTox [49] and HepaTox [50].

Since syndrome differentiation and individualized treatment are main features of TCM, excessive use of CHM is necessary and universal sometimes. It is also essential to further analyze its relationship with hepatotoxicity on the basis of more data. In our results, the values of liver tests were relatively high, which may be related to our strict inclusion criteria. The majority of cases were grouped into levels of highly probable or probable, indicating a high quality of causality assessment. What is more, many articles included did not provide a score of RUCAM, while the tool is widely accepted by researchers and clinicians [51–53]. The RUCAM scale is not without problems, especially for CHM induced liver injury. Previous information of CHM is not always available, and contamination by heavy metals and adulteration also provide challenges [54], which should be clarified before causality assessment. But it at least provides us with a framework, within which clinicians can organize the history taking and laboratory tests.

Based on our work, some suggestions were offered. First, CHM identified with reported hepatotoxicity should be used carefully. Secondly, since there is uncertain accuracy in determining the relationship between CHM and liver injury in most reports, we would like to adopt a strict definition of hepatotoxicity and the RUCAM scale, which contributes to excluding cases of other causes and offers causality evidence. Thirdly, when clinicians and researchers plan to report cases or carry out associated studies, it is advisable that details of causative herbs or patent drugs, like locality, botanical classification, brand names, detailed herbal ingredients and contents, and usage and dosage, should be provided at full length. What is more, it is necessary to build up a new website that provides up-to-date, comprehensive, and unbiased information about CHM induced liver injury and standardizes submission of associated information. Finally, since CHM is general designation of various kinds of herbs, we consider it improper to compare CHM with a single medicine.

In contrast with previous reviews, we adopted a high threshold of laboratory tests to avoid nonspecific liver injuries and included electronic data in China, which explained the difference from results of other reviews. Also, due to much more attention attracted in recent years, the increasing number of articles [7], and the development of diagnosis [55], our review focused on publications of the past 5 years. Of course, our review has its limitations. Above all, gray literature was not included, since cases unpublished were unavailable. A large number of publications searched failed to provide essential information associated with our inclusion criteria, which leads to missing data and may result in a risk of bias. Moreover, detailed information of herbs in the primary articles, like plant family, subfamily, species, subspecies, and locality, was unmentioned, which contributes to excluding adulteration and contamination. Finally, while the strict definition of hepatotoxicity helps us exclude unspecific liver injury, it may cause some toxic herbs to be missed. In fact, numbers of articles, especially articles in Chinese, were excluded for a different standard of diagnosis with ours.

In conclusion, CHM, especially He-Shou-Wu and those for dermatosis and osteoarthrosis, should be used carefully, and routine liver tests may be needed. Although cases have been increasingly reported, details about causative herbs need to be particularly illustrated. Liver injury from CHM is similar to that from conventional medicines in patent period, injury pattern, and prognosis. Further studies are needed on toxicity mechanisms and biomarkers, and more RUCAM should be used in future cases.

## Supplementary Material

Detailed information about all included cases was supplied in the supplementary table, including causative herbs, demographic information, regional distribution, usage and dosage, latent period, laboratory results, pattern of liver injury, causality assessment, reexposure results, and clinical outcomes.

## Figures and Tables

**Table 1:** List of CHM with reported hepatotoxicity.

CHM	Part used/ingredients	Potential toxicity mechanism	Application	Recommended dosage	Cases	References
He-Shou-Wu	Radix	Probably associated with anthraquinone derivatives, lipid peroxidation, or an immune response [32, 33]	Alopecia and grey hair	3~6 g/d (unprocessed herb), 6~12 g/d (processed herb)	Preparation unmentioned, 25	Jung et al., 2011 [18]
					4 unprocessed, 14 processed	Dong et al., 2014 [19]
					1 unprocessed	Zhang et al., 2014 [27]
					Preparation unmentioned, 2	Yuan and Chen, 2014 [25]
					Preparation unmentioned, 1	Yang et al., 2014 [28]
					Preparation unmentioned, 18	Ren and Xu, 2015 [29]

Cang-Er-Zi	Fruit	Probably kaurene glycosides induced liver injury via oxidative stress as lipid peroxidation in liver [34]	To eliminate wind and dampness	3~10 g/d	1	Wang et al., 2013 [24]

Huang-Yao-Zi	Rhizoma	Probably oxidative stress injury caused by diosbulbin [35]	Thyroid nodule	4.5~9 g/d	1	Jiang and Yang, 2014 [26]

Lei-Gong-Teng	Radix	Probably associated with triptolide, lipid peroxidation, and an immune response [36, 37]	Osteoarthrosis	NA	4	Ren and Xu, 2015 [29]

Yang Xue Sheng Fa Jiao Nang	Shu-Di-Huang, Dang-Gui, Qiang-Huo (*Notopterygium incisum* Ting ex H. T. Chang), Mu-Gua (*Chaenomeles sinensis* (Thouin) Koehne), Chuan-Xiong, Bai-Shao (*Paeonia lactiflora* Pall.), Tu-Si-Zi (*Cuscuta chinensis* Lam.), Tian-Ma (*Gastrodia elata *Blume), Zhi-Shou-Wu		Alopecia	4 granules, twice a day	4	Ren and Xu, 2015 [29]

Bai Dian Feng Jiao Nang	Bu-Gu-Zhi 33.33 g, Huang-Qi (*Astragalus propinquus* Schischkin) 33.33 g, Hong-Hua 33.33 g, Chuan-Xiong 33.33 g, Dang-Gui 33.33 g, Xiang-Fu (*Cyperus rotundus* L.) 33.33 g, Tao-Ren (*Prunus persica* (L.) Batsch) 33.33 g, Dan-Shen (*Salvia miltiorrhiza* Bunge) 33.33 g, Wu-Shao-She (*Zaocys*) 33.33 g, Zi-Cao (*Lithospermum erythrorhizon* Siebold & Zucc.) 33.33 g, Bai-Xian-Pi 33.33 g, Shan-Yao (*Dioscorea oppositifolia* L.) 33.33 g, Gan-Jiang 33.33 g, Long-Dan (*Gentiana scabra* Bunge) 33.33 g, Yan-Ji-Li (*Tribulus terrestris* L.) 433.33 g		Leucoderma	3~4 granules, twice a day	4	Ren and Xu, 2015 [29]

Xiao Yin Pian	Di-Huang, Dan-Pi (*Paeonia officinalis* L.), Chi-Shao, Dang-Gui, Ku-Shen (*Sophora flavescens* Aiton), Jin-Yin-Hua (*Lonicera japonica* Thunb.), Xuan-Shen (*Scrophularia ningpoensis* Hemsl.), Niu-Bang-Zi (*Arctium lappa* L.), Chan-Tui (*Cicadae periostracum*), Bai-Xian-Pi, Fang-Feng (*Saposhnikovia divaricata* (Trucz.) Schischk.), Da-Qing-Ye (*Wrightia laevis* Hook. f.), Hong-Hua		Psoriasis	5~7 tablets, three times a day	3	Ren and Xu, 2015 [29]

Qu Bai Ba Bu Pian	—		Leucoderma	NA	2	Ren and Xu, 2015 [29]

Bu Shen Sheng Fa Tang	—		Alopecia	NA	1	Ren and Xu, 2015 [29]

Ze Qi Chong Ji	—		Psoriasis	NA	1	Ren and Xu, 2015 [29]

Xian Ling Gu Bao Jiao Nang	—		Osteoarthrosis	NA	3	Ren and Xu, 2015 [29]

Gu Kang Jiao Nang	—		Osteoarthrosis	NA	2	Ren and Xu, 2015 [29]

Zhuang Gu Jiao Nang	—		Osteoarthrosis	NA	1	Ren and Xu, 2015 [29]

Ling Zhi Yi Shou Jiao Nang	—		Health promotion	NA	2	Ren and Xu, 2015 [29]

Ling Zhi Jiao Nang	—		Health promotion	NA	1	Ren and Xu, 2015 [29]

Hui Chun Ru Yi Jiao Nang	—		Symptoms like dizziness, a poor memory, fatigue, tinnitus, and soreness in the lower back and knees	NA	1	Ren and Xu, 2015 [29]

Ru Bi San	—		Hyperplasia of mammary gland	NA	1	Ren and Xu, 2015 [29]

ShuXiong Jiao Nang	San-Qi (*Panax pseudoginseng* Wall. var. *notoginseng* (Burkill) Hoo et Tseng) 166.7 g, Hong-Hua 166.7 g, Chuan-Xiong 333.3 g		Hyperplasia of mammary gland	3 granules, three times a day	1	Ren and Xu, 2015 [29]

Zeng Sheng Ping	—		Tumor	NA	1	Ren and Xu, 2015 [29]

Long Bi Shu	Bu-Gu-Zhi, Yi-Mu-Cao (*Leonurus artemisia* (Lour.) S. Y. Hu), Jin-Qian-Cao (*Lysimachia christinae* Hance), Hai-Jin-Sha (*Lygodium japonicum* (Thunb.) Sw.), Amber, Shan-Ci-Gu (*Iphigenia indica* Kunth)		Hyperplasia of prostate gland	3 granules, twice a day	4	Ren and Xu, 2015 [29]

Zhi Xue Jiao Nang	—		Hemorrhoids	NA	2	Ren and Xu, 2015 [29]

Move Free	Containing glucosamine, chondroitin, methylsulfonylmethane, black catechu, maltodextrin, Huang-Qin (*Scutellaria baicalensis* Georgi)		Arthritis	NA	1	Yang et al., 2012 [21]
					1	Dhanasekaran et al., 2013 [17]

Ban Tu Wan	Di-Huang, Shu-Di-Huang, Zhi-Shou-Wu, Dang-Gui, Dan-Shen, Bai-Shao, Wu-Wei-Zi (*Schisandra chinensis* (Turcz.) Baill.), Qiang-Huo, Mu-Gua		Alopecia	5 g, three times a day	1	Cortez et al., 2012 [16]

Kamishoyosan	Chai-Hu (*Bupleurum chinense* DC.), Dan-Pi, Bai-Zhu (*Atractylodes macrocephala* Koidz.), Ri-Ben-Dang-Gui (*Angelica acutiloba* (Sieb. et Zucc.) Kitagawa), Fu-Ling (*Poria cocos* (Schw.) Wolf.), Zhi-Zi (*Gardenia jasminoides* J. Ellis), Bai-Shao, Sheng-Jiang (*Zingiber officinale* Roscoe), Gan-Cao (*Glycyrrhiza uralensis* Fisch.), Bo-He (*Mentha haplocalyx* Briq.)		Postmenopausal syndrome	NA	1	Inoue et al., 2011 [23]

Qi Bao Mei Ran Wan	Zhi-Shou-Wu, Dang-Gui, Bu-Gu-Zhi, Gou-Qi (*Lycium chinense* Mill.), Tu-Si-Zi, Fu-Ling, Niu-Xi (*Achyranthes bidentata* Blume)		Grey hair	NA	1	Li et al., 2015 [15]

Herbal extracts containing Hu-Ji-Sheng and Ye-Ge			Health promotion	NA	1	Kim et al., 2015 [22]

Herbal tea containing Kelp			Type 2 diabetes mellitus	NA	1	Viswanathan and Patel, 2013 [20]

*Decoction*: Lian-Qiao (*Forsythia suspensa* (Thunb.) Vahl) 10 g, Pu-Gong-Ying (*Taraxacum mongolicum* Hand.-Mazz.) 10 g, Zi-Hua-Di-Ding (*Viola philippica* Cav.) 10 g, Ye-Ju-Hua (*Chrysanthemum indicum* L.) 10 g, Bai-Zhi (*Angelica dahurica* (Fisch. ex Hoffm.) Benth. et Hook. f. ex Franch. et Sav.) 10 g, Huang-Qin 10 g, Xuan-Shen 10 g, Gan-Cao 10 g, Sheng-Shi-Gao (*Gypsum fibrosum*) 15 g, Dan-Pi 10 g, Bai-Xian-Pi 10 g, Bai-Mao-Gen (*Imperata cylindrica* (L.) Raeusch.) 10 g, Yan-Ji-Li 10 g, Tao-Ren 10 g, Hong-Hua 10 g, Dong-Gua-Pi (*Benincasa hispida* (Thunb.) Cogn.) 10 g, Di-Gu-Pi (*Lycium chinense* Mill.) 10 g, Di-Fu-Zi (*Kochia scoparia* (L.) Schrad.) 10 g, Sheng-Bai-Zhu 10 g, Chuan-Xiong 10 g, Fu-Ling-Pi 10 g, Fa-Ban-Xia (*Pinellia ternata* (Thunb.) Makino) 10 g, Chen-Pi (*Citrus reticulata* Blanco) 10 g, Tu-Fu-Ling (*Smilax glabra* Roxb.) 10 g			Eczema	NA	1	Mao et al., 2013 [30]

*Pi Fu Bing Xue Du Wan and decoction*: Chai-Hu, Dang-Gui, Bai-Zhu, Fu-Ling, Bo-He, Wu-Mei *(Prunus mume* (Siebold) Siebold & Zucc.), Fang-Feng, Yin-Chai-Hu (*Stellaria dichotoma* L. var. *lanceolata* Bge.), Wu-Wei-Zi, Pi-Pa-Ye (*Eriobotrya japonica* (Thunb.) Lindl.), Chuan-Shan-Jia (*Manis squama*), Ze-Xie (*Alisma plantago-aquatica* Linn.), He-Shou-Wu, Lu-Lu-Tong (*Liquidambar formosana* Hance), Xu-Duan (*Dipsacus inermis* Wall.), Nv-Zhen-Zi (*Ligustrum lucidum* Ait.), Han-Lian-Cao (*Eclipta prostrata* (L.) L.), Gui-Zhi (*Cinnamomum cassia *Presl)			Psoriasis	NA	1	Wei, 2011 [31]

NA = not available.

**Table 2:** Clinical information of included cases with highly probable or probable causality grading.

CHM	Sex/age (y)	Usage and dosage	Exposure	ALT (IU/L)/AST (IU/L)/ TBIL (mg/dl)/ALP (IU/L)	Pattern	RUCAM grade (scores)	Outcome	Reexposure	Countries
Sheng-He-Shou-Wu	M/45	1 kg soaked in 2.5 kg wine, 25 mL/day	7 d	2870/2599/2.26/109	Hepatocellular	Highly probable (13)	Recovery	NA	China
Sheng-He-Shou-Wu	M/63	1 kg soaked in 22 kg wine, 250 mL/day	15 d	601/1515/2.87/181	Hepatocellular	Highly probable (11)	Recovery	NA	China
He-Shou-Wu	M/29	100 g consumed as tea, 100 mL/day	29 d	1792/899/5.51/138	Hepatocellular	Highly probable (11)	Recovery	NA	China
He-Shou-Wu	M/40	1 kg soaked in 10 kg wine, 300 mL/day	28 d	878/853/7.47/112	Hepatocellular	Highly probable (10)	Recovery	NA	China
He-Shou-Wu	M/40	1 kg soaked in 20 kg alcohol, 50 mL/day	43 d	4095/2473/1.7/220	Hepatocellular	Highly probable (10)	Recovery	NA	China
He-Shou-Wu	M/55	1 kg soaked in 15 kg alcohol, 300 mL/day	4 d	1056/769/10.29/176	Hepatocellular	Highly probable (10)	Recovery	NA	China
He-Shou-Wu	M/61	NA	1 d	818/NA/1.77/109	Hepatocellular	Highly probable (10)	Recovery	Positive	Korea
He-Shou-Wu	M/61	NA	60 d	885/NA/21.2/224	Mixed	Highly probable (10)	Recovery	NA	Korea
Sheng-He-Shou-Wu	M/40	1 kg soaked in 10 kg wine, 200 mL/day	1 d	3120/1615/16.78/151	Hepatocellular	Highly probable (10)	Recovery	NA	China
Move Free	F/78	a recommended dose of 1 tablet twice a day	21 d	1626/1053/7.2/354	Hepatocellular	Highly probable (10)^†^	Recovery	NA	USA
He-Shou-Wu	F/45	Not available	7 d	1196/507/0.88/816	Hepatocellular	Highly probable (9)	Recovery	NA	China
He-Shou-Wu	M/31	10 g powder consumed directly, 20 g/day	14 d	2026/858/2.19/180	Hepatocellular	Highly probable (9)	Recovery	NA	China
He-Shou-Wu	M/56	1 slice consumed as tea, 100 mL/day	120 d	2313/1202/5.81/305	Hepatocellular	Highly probable (9)	Recovery	NA	China
He-Shou-Wu	F/37	1 tablespoon consumed as decoction, 250 mL/day	52 d	922/319/9.06/116	Hepatocellular	Highly probable (9)	Recovery	NA	China
He-Shou-Wu	F/46	1 tablespoon consumed as decoction, 250 mL/day	50 d	1127/297/1.94/203	Hepatocellular	Highly probable (9)	Recovery	NA	China
He-Shou-Wu	F/18	1 tablespoon consumed as decoction, 100 mL/day	67 d	1074/348/0.8/89	Hepatocellular	Highly probable (9)	Recovery	NA	China
He-Shou-Wu	M/40	3 tablespoons consumed as decoction, 100 mL/day	23 d	1987/872/11/182	Hepatocellular	Highly probable (9)	Recovery	NA	China
He-Shou-Wu	M/34	NA	30 d	1452/NA/25.3/111	Hepatocellular	Highly probable (9)	Recovery	NA	Korea
He-Shou-Wu	M/58	NA	35 d	1898/NA/13.4/134	Hepatocellular	Highly probable (9)	Recovery	NA	Korea
He-Shou-Wu	M/45	NA	30 d	1400/NA/2.04/125	Hepatocellular	Highly probable (9)	Recovery	NA	Korea
He-Shou-Wu	M/49	NA	90 d	1235/NA/32.9/465	Mixed	Highly probable (9)	Recovery	NA	Korea
He-Shou-Wu	M/46	NA	2 d	1287/NA/19.7/146	Hepatocellular	Highly probable (9)	Recovery	NA	Korea
He-Shou-Wu	M/62	NA	90 d	1174/NA/9.2/175	Hepatocellular	Highly probable (9)	Recovery	NA	Korea
He-Shou-Wu	F/63	NA	30 d	943/NA/4.2/137	Hepatocellular	Highly probable (9)	Recovery	NA	Korea
He-Shou-Wu	M/44	NA	7 d	1077/NA/15/197	Hepatocellular	Highly probable (9)	Recovery	NA	Korea
Herbal extracts containing Hu-Ji-Sheng and Ye-Ge	M/55	NA	30 d	1528/1108/6.3/160	Hepatocellular	Highly probable (9)	Recovery	NA	Korea
He-Shou-Wu	M/38	2 slices consumed as tea, 500 mL/day	1 d	1815/1351/5.3/108	Hepatocellular	Probable (8)	Recovery	NA	China
He-Shou-Wu	M/48	100 g mixed with 150 g bee honey, 8 g/day	10 d	1891/798/7.9/159	Hepatocellular	Probable (8)	Recovery	NA	China
He-Shou-Wu	F/61	10–20 g/d	60 d	56/61/28.07/601	Cholestatic	Probable (8)^†^	Recovery	NA	China
He-Shou-Wu	M/57	NA	30 d	853/NA/28.1/173	Mixed	Probable (8)	Death	NA	Korea
He-Shou-Wu	F/47	NA	60 d	1947/NA/30.4/218	Hepatocellular	Probable (8)	Recovery	NA	Korea
He-Shou-Wu	M/59	NA	30 d	1245/NA/1.6/155	Hepatocellular	Probable (8)	Recovery	NA	Korea
He-Shou-Wu	F/46	NA	30 d	1804/NA/6.2/81	Hepatocellular	Probable (8)	Recovery	NA	Korea
He-Shou-Wu	M/45	NA	20 d	271/NA/2.9/164	Mixed	Probable (8)	Recovery	NA	Korea
He-Shou-Wu	M/65	NA	10 d	1107/NA/21.9/197	Hepatocellular	Probable (8)	Recovery	NA	Korea
He-Shou-Wu	F/42	NA	10 d	500/NA/1.6/181	Mixed	Probable (8)	Recovery	NA	Korea
He-Shou-Wu	M/42	NA	60 d	1706/NA/26.3/147	Hepatocellular	Probable (8)	Recovery	NA	Korea
He-Shou-Wu	F/48	NA	3 d	1142/NA/15.9/145	Hepatocellular	Probable (8)	Recovery	NA	Korea
Sheng-He-Shou-Wu	M/50	1 kg soaked in 5 kg wine, 500 mL/day	20 d	1992/1155/6.73/185	Hepatocellular	Probable (8)	Recovery	NA	China
Qi Bao Mei Ran Wan	M/26	Taken at the recommended dosages	30 d	1674/617/3.2/normal	Hepatocellular	Probable (8)^†^	Recovery	NA	China
He-Shou-Wu	F/41	15 tablets per day	21 d	104/85/21.7/947	Cholestatic	Probable (7)^†^	Recovery	NA	China
He-Shou-Wu	M/37	1 tablespoon consumed as decoction, 1500 mL/day	7 d	1613/835/2.26/177	Hepatocellular	Probable (7)	Recovery	NA	China
He-Shou-Wu	F/54	NA	4 d	1752/NA/8.4/286	Hepatocellular	Probable (7)	Recovery	NA	Korea
He-Shou-Wu	M/24	NA	60 d	1652/NA/31.9/140	Hepatocellular	Probable (7)	Liver transplantation	NA	Korea
He-Shou-Wu	M/42	NA	120 d	1677/NA/15.8/93	Hepatocellular	Probable (7)	Recovery	NA	Korea
He-Shou-Wu	M/41	NA	30 d	520/NA/9.9/143	Mixed	Probable (7)	Recovery	NA	Korea
Huang-Yao-Zi	F/66	30 g decocted in water for oral dose per day	21 d	1042/1006/16.44/normal	Hepatocellular	Probable (7)^†^	Recovery	NA	China
He-Shou-Wu YanShouPian	M/17	15 tablets per day	45 d	1501/545/18.63/155	Hepatocellular	Probable (6)^†^	Recovery	NA	China
He-Shou-Wu	F/54	NA	180 d	1519/NA/11.7/187	Hepatocellular	Probable (6)	Recovery	NA	Korea
He-Shou-Wu/1	M/53	NA	180 d	1227/NA/33.2/370	Mixed	Probable (6)	Recovery	NA	Korea
Herbal tea containing Kelp	F/40	3 cups per day	60 d	435/219/9.2/435	Mixed	Probable (6)^†^	Recovery	NA	USA
Kamishoyosan	F/48	NA	60 d	972/900/12.8/420	Hepatocellular	Probable (6)^†^	Recovery	NA	Japan
Move Free	F/62	4 tablets/d for two and a half weeks tapered down to 2 tablets/d for four days	21 d	1247/893/6.9/297	Hepatocellular	Probable (6)^†^	Recovery	NA	USA
Decoction [30]	F/51	Brewed with water, two times a day	23 d	758/1262/9.92/normal	Hepatocellular	Probable (6)^†^	Recovery	NA	China

NA = not available, ALT = alanine aminotransferase, AST = aspartate aminotransferase, TB = total bilirubin, and ALP = alkaline phosphatase. ^†^The article did not provide an outcome of RUCAM scale but detailed information based on which a score was given.
