# Supplementary material for: Systematic Review on Chinese Herbal Medicine Induced Liver Injury
Source: Evid Based Complement Alternat Med. 2016 Aug 29;2016:3560812. doi: 10.1155/2016/3560812 (PMC5019919; doi:10.1155/2016/3560812)
Supplement: Supplementary file 1 — Detailed information about all included cases was supplied in the supplementary table, including causative herbs, demographic information, regional distribution, usage and dosage, latent period, laboratory results, pattern of liver injury, causality assessment, reexposure results, and clinical outcomes. [file 3560812.f1.docx]

| **Supplementary Table. Clinical information of all included cases** | | | | | | | | | |
| --- | --- | --- | --- | --- | --- | --- | --- | --- | --- |
| **Chinese herbal medicine/cases** | **Sex/age(y)** | **Usage and dosage** | **Exposure** | **ALT(IU/L)/AST(IU/L)/TBIL(mg/dl)/ALP(IU/L)** | **Pattern** | **RUCAM grade (scores)** | **Outcome** | **Reexposure** | **Countries** |
| **Sheng-He-Shou-Wu /1** | M/63 | 1 kg soaked in 22 kg wine,250 mL/day | 15d | 601/1515/2.87/181 | Hepatocellular | Highly probable (11) | Recovery | NA | China |
| **Sheng-He-Shou-Wu /1** | M/45 | 1 kg soaked in 2.5 kg wine,25 mL/day | 7d | 2870/2599/2.26/109 | Hepatocellular | Highly probable (13) | Recovery | NA | China |
| **Sheng-He-Shou-Wu /1** | M/40 | 1 kg soaked in 10 kg wine,200 mL/day | 1d | 3120/1615/16.78/151 | Hepatocellular | Highly probable (10) | Recovery | NA | China |
| **Sheng-He-Shou-Wu /1** | M/50 | 1 kg soaked in 5 kg wine,500 mL/day | 20d | 1992/1155/6.73/185 | Hepatocellular | Probable (8) | Recovery | NA | China |
| **He-Shou-Wu /1** | M/40 | 1 kg soaked in 10 kg win,300 mL/day | 28d | 878/853/7.47/112 | Hepatocellular | Highly probable (10) | Recovery | NA | China |
| **He-Shou-Wu /1** | F/37 | 1 tablespoon consumed as a decoction, 250 mL/day | 52d | 922/319/9.06/116 | Hepatocellular | Highly probable (9) | Recovery | NA | China |
| **He-Shou-Wu /1** | F/46 | 1 tablespoon consumed as a decoction,250 mL/day | 50d | 1127/297/1.94/203 | Hepatocellular | Highly probable (9) | Recovery | NA | China |
| **He-Shou-Wu /1** | F/18 | 1 tablespoon consumed as a decoction,100 mL/day | 67d | 1074/348/0.8/89 | Hepatocellular | Highly probable (9) | Recovery | NA | China |
| **He-Shou-Wu /1** | M/37 | 1 tablespoon consumed as a decoction,1500 mL/day | 7d | 1613/835/2.26/177 | Hepatocellular | Probable (7) | Recovery | NA | China |
| **He-Shou-Wu /1** | M/40 | 3 tablespoon consumed as a decoction,100 mL/day | 23d | 1987/872/11/182 | Hepatocellular | Highly probable (9) | Recovery | NA | China |
| **He-Shou-Wu /1** | M/29 | 100 g consumed as tea,100 mL/day | 29d | 1792/899/5.51/138 | Hepatocellular | Highly probable (11) | Recovery | NA | China |
| **He-Shou-Wu /1** | M/40 | 1 kg soaked in 20 kg alcohol,50 mL/day | 43d | 4095/2473/1.7/220 | Hepatocellular | Highly probable (10) | Recovery | NA | China |
| **He-Shou-Wu /1** | F/45 | Not available | 7d | 1196/507/0.88/816 | Hepatocellular | Highly probable (9) | Recovery | NA | China |
| **He-Shou-Wu /1** | M/38 | 2 slices consumed as tea，500 mL/day | 1d | 1815/1351/5.3/108 | Hepatocellular | Probable (8) | Recovery | NA | China |
| **He-Shou-Wu /1** | M/55 | 1 kg soaked in 15 kg alcohol，300 mL/day | 4d | 1056/769/10.29/176 | Hepatocellular | Highly probable (10) | Recovery | NA | China |
| **He-Shou-Wu /1** | M/48 | 100 g mixed with 150 g bee honey，8 g/day | 10d | 1891/798/7.9/159 | Hepatocellular | Probable (8) | Recovery | NA | China |
| **He-Shou-Wu /1** | M/31 | 10 g powder consumed directly,20 g/day | 14d | 2026/858/2.19/180 | Hepatocellular | Highly probable (9) | Recovery | NA | China |
| **He-Shou-Wu /1** | M/56 | 1 slice consumed as tea,100 mL/day | 120d | 2313/1202/5.81/305 | Hepatocellular | Highly probable (9) | Recovery | NA | China |
| **Sheng-He-Shou-Wu /1** | F/48 | 5 g mixed with water every two days, about 200 g in total | 60d | 349.4/1339.4/16.43/normal | Hepatocellular | Possible (4)^†^ | Recovery | NA | China |
| **He-Shou-Wu / 18** | NA | NA | NA | NA | NA | ≥3 | NA | NA | China |
| **He-Shou-Wu /1** | F/61 | 10-20g/d | 60d | 56/61/28.07/601 | Cholestatic | Probable (8)^†^ | Recovery | NA | China |
| **He-Shou-Wu /1** | M/57 | NA | 30d | 853/NA/28.1/173 | Mixed | Probable (8) | Death | NA | Korea |
| **He-Shou-Wu /1** | M/49 | NA | 90d | 1235/NA/32.9/465 | Mixed | Highly probable (9) | Recovery | NA | Korea |
| **He-Shou-Wu /1** | M/46 | NA | 2d | 1287/NA/19.7/146 | Hepatocellular | Highly probable (9) | Recovery | NA | Korea |
| **He-Shou-Wu /1** | F/47 | NA | 60d | 1947/NA/30.4/218 | Hepatocellular | Probable (8) | Recovery | NA | Korea |
| **He-Shou-Wu /1** | M/34 | NA | 30d | 1452/NA/25.3/111 | Hepatocellular | Highly probable (9) | Recovery | NA | Korea |
| **He-Shou-Wu /1** | M/58 | NA | 35d | 1898/NA/13.4/134 | Hepatocellular | Highly probable (9) | Recovery | NA | Korea |
| **He-Shou-Wu /1** | M/59 | NA | 30d | 1245/NA/1.6/155 | Hepatocellular | Probable (8) | Recovery | NA | Korea |
| **He-Shou-Wu /1** | F/54 | NA | 4d | 1752/NA/8.4/286 | Hepatocellular | Probable (7) | Recovery | NA | Korea |
| **He-Shou-Wu /1** | F/46 | NA | 30d | 1804/NA/6.2/81 | Hepatocellular | Probable (8) | Recovery | NA | Korea |
| **He-Shou-Wu /1** | M/62 | NA | 90d | 1174/NA/9.2/175 | Hepatocellular | Highly probable (9) | Recovery | NA | Korea |
| **He-Shou-Wu /1** | F/63 | NA | 30d | 943/NA/4.2/137 | Hepatocellular | Highly probable (9) | Recovery | NA | Korea |
| **He-Shou-Wu /1** | M/24 | NA | 60d | 1652/NA/31.9/140 | Hepatocellular | Probable (7) | Liver Transplantation | NA | Korea |
| **He-Shou-Wu /1** | M/45 | NA | 20d | 271/NA/2.9/164 | Mixed | Probable (8) | Recovery | NA | Korea |
| **He-Shou-Wu /1** | M/41 | NA | 30d | 520/NA/9.9/143 | Mixed | Probable (7) | Recovery | NA | Korea |
| **He-Shou-Wu /1** | M/44 | NA | 7d | 1077/NA/15/197 | Hepatocellular | Highly probable (9) | Recovery | NA | Korea |
| **He-Shou-Wu /1** | M/65 | NA | 10d | 1107/NA/21.9/197 | Hepatocellular | Probable (8) | Recovery | NA | Korea |
| **He-Shou-Wu /1** | M/53 | NA | 180d | 1227/NA/33.2/370 | Mixed | Probable (6) | Recovery | NA | Korea |
| **He-Shou-Wu /1** | F/42 | NA | 10d | 500/NA/1.6/181 | Mixed | Probable (8) | Recovery | NA | Korea |
| **He-Shou-Wu /1** | M/61 | NA | 1d | 818/NA/1.77/109 | Hepatocellular | Highly probable (10) | Recovery | Positive | Korea |
| **He-Shou-Wu /1** | M/42 | NA | 120d | 1677/NA/15.8/93 | Hepatocellular | Probable (7) | Recovery | NA | Korea |
| **He-Shou-Wu /1** | F/48 | NA | 3d | 1142/NA/15.9/145 | Hepatocellular | Probable (8) | Recovery | NA | Korea |
| **He-Shou-Wu /1** | F/54 | NA | 180d | 1519/NA/11.7/187 | Hepatocellular | Probable (6) | Recovery | NA | Korea |
| **He-Shou-Wu /1** | M/61 | NA | 60d | 885/NA/21.2/224 | Mixed | Highly probable (10) | Recovery | NA | Korea |
| **He-Shou-Wu /1** | M/45 | NA | 30d | 1400/NA/2.04/125 | Hepatocellular | Highly probable (9) | Recovery | NA | Korea |
| **He-Shou-Wu /1** | M/42 | NA | 60d | 1706/NA/26.3/147 | Hepatocellular | Probable (8) | Recovery | NA | Korea |
| **He-Shou-Wu /1** | F/41 | 15 tablets per day | 21d | 104/85/21.7/947 | Cholestatic | Probable (7)^†^ | Recovery | NA | China |
| **He-Shou-Wu Yan Shou Pian/1** | M/17 | 15 tablets per day | 45d | 1501/545/18.63/155 | Hepatocellular | Probable (6)^†^ | Recovery | NA | China |
| **Cang-Er-Zi /1** | M/70 | 500 g powders mixed with water consumed in 2days | 15d | 3359/742/2.75/normal | Hepatocellular | Possible (5)^†^ | Recovery | NA | China |
| **Huang-Yao-Zi /1** | F/66 | 30 g decocted in water for oral dose per day | 21d | 1042/1006/16.44/normal | Hepatocellular | Probable (7)^†^ | Recovery | NA | China |
| **Yang Xue Sheng Fa Jiao Nang/4** | NA | NA | NA | NA | NA | ≥3 | NA | NA | China |
| **Bai Dian Feng Jiao Nang/4** | NA | NA | NA | NA | NA | ≥3 | NA | NA | China |
| **Xiao Yin Pian/3** | NA | NA | NA | NA | NA | ≥3 | NA | NA | China |
| **Qu Bai Ba Bu Pian/2** | NA | NA | NA | NA | NA | ≥3 | NA | NA | China |
| **Bu Shen Sheng Fa Tang/1** | NA | NA | NA | NA | NA | ≥3 | NA | NA | China |
| **Ze Qi Chong Ji/1** | NA | NA | NA | NA | NA | ≥3 | NA | NA | China |
| **Lei-Gong-Teng /4** | NA | NA | NA | NA | NA | ≥3 | NA | NA | China |
| **Xian Ling Gu Bao Jiao Nang/3** | NA | NA | NA | NA | NA | ≥3 | NA | NA | China |
| **Gu Kang Jiao Nang/2** | NA | NA | NA | NA | NA | ≥3 | NA | NA | China |
| **Zhuang Gu Jiao Nang/1** | NA | NA | NA | NA | NA | ≥3 | NA | NA | China |
| **Ling Zhi Yi Shou Jiao Nang/2** | NA | NA | NA | NA | NA | ≥3 | NA | NA | China |
| **Ling Zhi Jiao Nang/1** | NA | NA | NA | NA | NA | ≥3 | NA | NA | China |
| **Hui Chun Ru Yi Jiao Nang/1** | NA | NA | NA | NA | NA | ≥3 | NA | NA | China |
| **Ru Bi San/1** | NA | NA | NA | NA | NA | ≥3 | NA | NA | China |
| **Shu Xiong Jiao Nang/1** | NA | NA | NA | NA | NA | ≥3 | NA | NA | China |
| **Zeng Sheng Ping/1** | NA | NA | NA | NA | NA | ≥3 | NA | NA | China |
| **Long Bi Shu/4** | NA | NA | NA | NA | NA | ≥3 | NA | NA | China |
| **Zhi Xue Jiao Nang/2** | NA | NA | NA | NA | NA | ≥3 | NA | NA | China |
| **Move Free/1** | F/62 | 4 tablets/d for two and a half weeks and had tapered down to 2 tablets/d for four days | 21d | 1247/893/6.9/297 | Hepatocellular | Probable (6)^†^ | Recovery | NA | USA |
| **Move Free/1** | F/78 | a recommended dose of 1 tablet twice a day | 21d | 1626/1053/7.2/354 | Hepatocellular | Highly probable(10)^†^ | Recovery | NA | USA |
| **Ban Tu Wan/1** | F/NA | NA | 60-90d | 5386/NA/4.1/199 | Hepatocellular | Possible (3)^†^ | Death | NA | USA |
| **Kamishoyosan/1** | F/48 | NA | 60d | 972/900/12.8/420 | Hepatocellular | Probable (6)^†^ | Recovery | NA | Japan |
| **Qi Bao Mei Ran Wan /1** | M/26 | Taken at the recommended dosages | 30d | 1674/617/3.2/normal | Hepatocellular | Probable (8)^†^ | Recovery | NA | China |
| **Herbal Extracts Containing Hu-Ji-Sheng and Ye-Ge /1** | M/55 | NA | 30d | 1528/1108/6.3/160 | Hepatocellular | Highly probable(9) | Recovery | NA | Korea |
| **Herbal Tea Containing Kelp/1** | F/40 | 3 cups per day | 60d | 435/219/9.2/435 | Mixed | Probable (6)^†^ | Recovery | NA | USA |

NA=not available, ALT= alanine aminotransferase, AST= aspartate aminotransferase, TB= total bilirubin, ALP= alkaline phosphatase, †=the article didn’t provide an outcome of CIOMS scale but detailed information based on which a score was given.
